# Neurodegeneration: Etiologies and New Therapies

**DOI:** 10.1155/2015/272630

**Published:** 2015-02-03

**Authors:** E. K. Tan, Amit K. Srivastava, W. David Arnold, Mahendra P. Singh, Yiying Zhang

**Affiliations:** ^1^Department of Neurology, Singapore General Hospital, National Neuroscience Institute and Duke NUS Graduate Medical School, Singapore 169857; ^2^Russell H. Morgan Department of Radiology and Radiological Science and Institute for Cell Engineering, The Johns Hopkins University School of Medicine, Baltimore, MD 21205, USA; ^3^Division of Neuromuscular Medicine, Department of Neurology, The Ohio State University, Columbus, OH 43210, USA; ^4^Department of Physical Medicine and Rehabilitation, The Ohio State University, Columbus, OH 43210, USA; ^5^Toxicogenomics and Predictive Toxicology Division, CSIR-Indian Institute of Toxicology Research, Lucknow 226 001, Uttar Pradesh, India; ^6^Department of Anesthesia, Critical Care and Pain Medicine, Massachusetts General Hospital, Harvard Medical School, Charlestown, MA 02129, USA

Neurodegeneration refers to the progressive loss of structure or function of neurons and it can lead to devastating neurological conditions such as Alzheimer's disease (AD), Parkinson's disease (PD), Huntington's disease (HD), and amyotrophic lateral sclerosis (ALS). Neurodegeneration is frequently multifactorial in origin though aging and genetic and environmental factors are thought to play a significant role. The relative contribution of genes and environmental factors has been debated. For AD and PD, both familial (monogenic and complex inheritance) and sporadic forms exist while some like HD are purely genetic in nature.

The key pathological hallmarks of neurodegenerative diseases include oxidative stress, proteasomal impairment, mitochondrial dysfunction, and accumulation of abnormal protein aggregates. Advances in the field of etiology and therapy are mainly based on understanding of basic biochemical and molecular events underlying degeneration in human postmortem brain specimens and animal models. Molecular imaging and the emergence of complex bioinformatics tools have provided important insights [1].

This special issue on neurodegeneration provides a platform for critical reviews on recent advances and original articles that offer significant insights into biochemical and molecular aspects of neurodegeneration with the potential of identifying novel therapeutic targets (such as synthetic/naturally occurring agents/extracts).

Oxidative stress, mitochondrial dysfunction, and gliosis are found to play critical functions in the process of neurodegeneration [2]. Epileptic seizures can vary from brief and undetectable reaction to prolonged and vigorous shaking. While the contributors of epilepsy are relatively mysterious, brain injury leading to neurodegeneration could also contribute to epileptic episodes. S. Puttachary et al. review the role of seizures-induced oxidative stress in neurodegeneration along with spontaneous recurrent seizures in temporal lobe epilepsy. They highlight the contribution of the mitochondrial dysfunction, gliosis, and neurodegeneration in temporal lobe epilepsy and alleviating effects of endogenous antioxidant pathways.

Although HD is an autosomal genetic disorder, development of a fully reliable rodent model that mimics all symptomatic and pathological traits of disease is still a challenge. Y. Mazurová et al. allude to the histopathological changes in the brain of transgenic HD rats. This paper emphasizes how glial cells could play a unique role and transgenic rats could be used as a valid model for diagnostic and therapeutic interventions.

The cardinal challenge in neurodegeneration is to either slow down or reverse disease progression. Extracts of naturally occurring agents theoretically may have less toxicity compared to synthetic drugs. F. Ghahremanitamadon et al. demonstrate the shielding efficacy of* Borago officinalis* extract in amyloid *β*-peptide (25–35)-induced oxidative stress and behavioral deficits. They demonstrate the usefulness of* Borago officinalis* extract in reducing memory impairment.

Another major difficulty is to effectively deliver an active or pro drug across the blood-brain barrier. J. Hou et al. describe an in vivo microdialysis of (N-[2-(4-hydroxy-phenyl)-ethyl]-2-(2,5-dimethoxy-phenyl)-3-(3-methoxy-4-22hydroxy-phenyl)-acrylamide) penetration through the blood-brain barrier in normal and 6-hydroxydopamine-induced parkinsonism. The findings could potentially lead to clinical trials of bioactive molecules.

Diagnosis and delineation of neurodegeneration are still complicated. A. P. Patterson et al. highlight the relevance of in vivo optical imaging systems in neurodegeneration. The authors draw attention to the contribution of imaging tools along with fluorescent and bioluminescent molecules, in the diagnosis and monitoring of neurodegenerative diseases.

The cause and effect of inflammation in neurodegeneration are still unknown. Y. Chao et al. provide a concise review of the involvement of inflammatory factors and immune system in the pathogenesis of PD and discuss the potential therapeutic targets to regulate immune response in the disease. In another review article, Y. Lee et al. described the role of microglia in pathogenesis of ischemic stroke and several therapeutic approaches to modulate microglial response.

Neurodegenerative processes are complex and clinical and pathological correlation is often hard to demonstrate. A multiprong research approach evaluating potential molecular targets, the most effective way to deliver potential pharmacological agents to the brain, and the appropriate design of clinical trials that can monitor and evaluate the efficacy of novel drugs will be vital. In this light, we hope this special issue can provide impetus for investigators to take on these challenges with the ultimate hope of finding a cure for many of the devastating neurological diseases.

*E. K. Tan* *Amit K. Srivastava* *W. David Arnold* *Mahendra P. Singh* *Yiying Zhang*
